# Tissue-Specific Regulation of Chromatin Insulator Function

**DOI:** 10.1371/journal.pgen.1003069

**Published:** 2012-11-29

**Authors:** Leah H. Matzat, Ryan K. Dale, Nellie Moshkovich, Elissa P. Lei

**Affiliations:** 1 Laboratory of Cellular and Developmental Biology, National Institute of Diabetes and Digestive and Kidney Diseases, National Institutes of Health, Bethesda, Maryland, United States of America; 2 Graduate Program in Molecular and Cell Biology, University of Maryland, College Park, Maryland, United States of America; The University of North Carolina at Chapel Hill, United States of America

## Abstract

Chromatin insulators organize the genome into distinct transcriptional domains and contribute to cell type–specific chromatin organization. However, factors regulating tissue-specific insulator function have not yet been discovered. Here we identify the RNA recognition motif-containing protein Shep as a direct interactor of two individual components of the *gypsy* insulator complex in *Drosophila*. Mutation of *shep* improves *gypsy*-dependent enhancer blocking, indicating a role as a negative regulator of insulator activity. Unlike ubiquitously expressed core *gypsy* insulator proteins, Shep is highly expressed in the central nervous system (CNS) with lower expression in other tissues. We developed a novel, quantitative tissue-specific barrier assay to demonstrate that Shep functions as a negative regulator of insulator activity in the CNS but not in muscle tissue. Additionally, mutation of *shep* alters insulator complex nuclear localization in the CNS but has no effect in other tissues. Consistent with negative regulatory activity, ChIP–seq analysis of Shep in a CNS-derived cell line indicates substantial genome-wide colocalization with a single *gypsy* insulator component but limited overlap with intact insulator complexes. Taken together, these data reveal a novel, tissue-specific mode of regulation of a chromatin insulator.

## Author Summary

Mounting evidence in human, mouse, and *Drosophila* demonstrates a role for the DNA–protein complexes known as chromatin insulators in orchestrating three-dimensional genome organization. Several genes that are only expressed in specific cell types display distinct chromatin configurations correlated with expression status. Recent evidence shows that chromatin insulators play a role in defining tissue-specific chromatin conformation; however, tissue-specific factors that may modulate insulator activity remain unknown. Here we identify a putative RNA–binding protein, Shep, which is expressed most highly in the CNS and interacts directly with insulator complexes. We developed a novel quantitative, tissue-specific insulator assay and found that Shep negatively regulates insulator activity in the CNS. We also find that mutation of *shep* alters insulator complex nuclear localization in the brain but not other tissues. Finally, we mapped Shep and *gypsy* insulator protein localization throughout the genome and found that Shep colocalizes with one individual insulator protein but less often than expected with an intact insulator complex. These data suggest that Shep negatively influences insulator activity in a tissue-specific manner.

## Introduction

Chromatin insulators are DNA-protein complexes that influence eukaryotic gene expression by organizing the genome into distinct transcriptional domains. Functionally conserved from *Drosophila* to humans, insulators regulate interactions between regulatory elements such as enhancers and promoters and demarcate silent and active chromatin regions (for review, see [1]). Chromatin insulators are thought to exert effects on gene expression by constraining the topology of chromatin and facilitating the formation of intra- and inter-chromosomal looping (for review, see [2]). These higher order interactions can vary between cell types, thereby facilitating tissue-specific transcriptional output.

*Drosophila* harbor several distinct classes of chromatin insulators, including the well studied *gypsy* insulator, also known as the Suppressor of Hairy wing (Su(Hw)) insulator. The zinc-finger DNA-binding protein, Su(Hw), recognizes a particular motif, imparting specificity to the *gypsy* insulator. In addition to Su(Hw), the core *gypsy* insulator complex contains Centrosomal protein 190 (CP190), which also harbors a zinc finger domain, and the non-DNA-binding protein, Modifier of mdg4 2.2 (Mod(mdg4)2.2). These core proteins are required for *gypsy* insulator activity [3]–[7]. Both CP190 and Mod(mdg4)2.2 contain broad complex, tramtrack, bric-a-brac (BTB) dimerization domains that have been suggested to mediate insulator-insulator interactions and facilitate the formation of long range insulator-mediated loops along the chromatin fiber [4], [8].

Specialized nuclear arrangement of *gypsy* insulator complexes correlates tightly with insulator function. The *gypsy* insulator proteins bind to thousands of sites throughout the genome with more than half of Su(Hw) binding sites occurring in intergenic regions and a large number of sites located within introns [9], [10]. Consistent with a role in boundary formation, Su(Hw) sites are positively correlated with both Lamin-associated domains and boundaries between transcriptionally active and silent chromatin [10], [11]. It has been shown that *gypsy* insulator proteins coalesce at a small number of foci in diploid nuclei, termed insulator bodies, which have been proposed to act either as hubs of higher order chromatin domains [8] or storage sites for insulator proteins [12]. Importantly, mutation of certain insulator components results in impaired insulator activity coincident with diffuse or smaller, more numerous insulator bodies [4], [8], [12]–[14]. However, formation of insulator bodies is not sufficient for *gypsy* insulator activity [15], [16], and a detailed mechanistic understanding of insulator bodies is still lacking. Nevertheless, the tight correlation between *gypsy* insulator function and insulator body localization suggests an important role for these structures. Finally, in addition to a variety of accessory proteins [17]–[19], a role for RNA in insulator function and insulator body organization was suggested based on RNA-dependent protein interaction with insulator complexes [20].

Genome-wide studies indicate that the locations of insulator protein binding sites are mainly consistent across different cell types but that insulator-dependent looping configurations may dictate differences in gene expression. In *Drosophila*, it has been shown that external stimuli can alter chromatin association of CP190, possibly leading to a change in chromatin looping [21]. Recent large-scale chromatin conformation capture (3C)-based studies have implicated insulator protein binding sites as key contact points mediating looping throughout the genome [22]–[25]. In several studies across species, specific chromatin conformations are observed in loci that produce tissue- or cell-type specific transcripts [26]–[32]. Whether insulators either establish tissue-specific chromatin organization or maintain configurations established via transcription is unclear. Furthermore, factors that control tissue-specific insulator-dependent chromatin organization remain unknown.

This study identifies a CNS enriched, RNA recognition motif (RRM) containing protein, Alan Shepard (Shep), as the first tissue-specific regulator of *gypsy* insulator activity and insulator body localization. We show that Shep interacts directly with Mod(mdg4)2.2 and Su(Hw) and also associates with *gypsy* insulator proteins *in vivo*. Using a novel quantitative, tissue-specific insulator assay, we find that Shep negatively regulates *gypsy* insulator activity in the CNS. In addition, mutation of Shep improves compromised insulator function and insulator body formation. Finally, genome-wide localization in the CNS-derived BG3 cell line reveals enrichment of overlap between Shep and Mod(mdg4)2.2 but less frequent than expected overlap among Shep, Su(Hw) and Mod(mdg4)2.2 together. These data suggest that *gypsy* chromatin insulator function can be regulated in a tissue-specific manner.

## Results

### Shep is a novel direct interactor of *gypsy* insulator complexes

**Figure 1:**
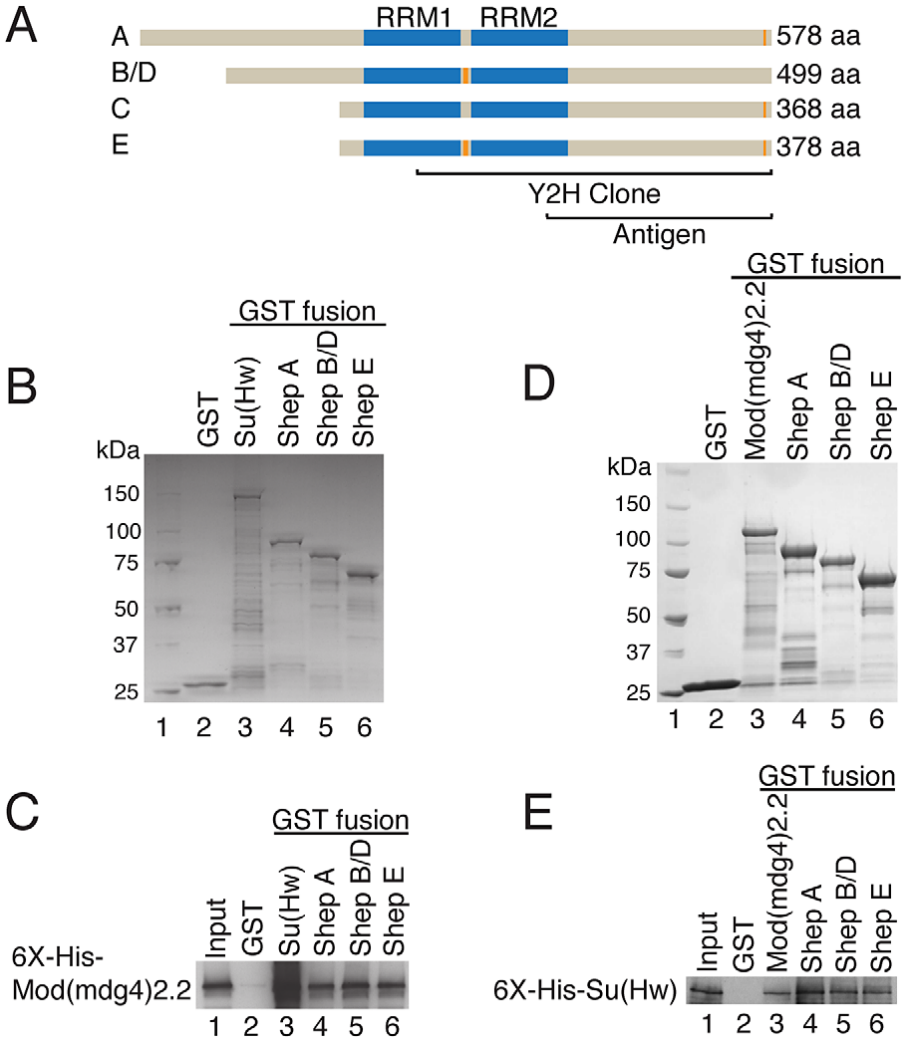
Shep associates directly with *gypsy* insulator complexes. (A) Diagram of Shep protein isoforms. RRMs (blue) and alternative amino acid stretches (not to scale, orange) are shown. Regions of Shep utilized for antibody production or contained in the yeast two-hybrid clone, which corresponds to exons present in isoform E, are indicated. (B) Coomassie staining of recombinant GST fusion proteins used for binding reactions in (C). Protein marker is run in lane 1. (C) Interaction of purified, soluble His-Mod(mdg4)2.2 (lane 1, 4.5% input) with immobilized GST (lane 2), GST-Su(Hw) (lane 3) or GST-Shep isoforms (lanes 4–6). Binding of His-Mod(mdg4)2.2 to GST-fusion proteins was detected by Western blotting. (D) Coomassie staining of recombinant GST fusion proteins used for binding reactions in (E). (E) Interaction of purified, soluble His-Su(Hw) (lane 1, 6.3% input) with immobilized GST (lane 2), GST-Mod(mdg4)2.2 (lane 3) or GST-Shep isoforms (lanes 4–6). Binding of His-Su(Hw) to GST-fusion proteins was detected by Western blotting.

The putative RNA-binding protein Shep was identified as a novel interaction partner of the *gypsy* insulator complex. Shep, encoded by the *alan shepard* locus, was found by yeast two-hybrid screening as a strong interactor of Mod(mdg4)2.2 [17; M. Capelson and V. Corces, personal communication]. The *shep* gene was named based on its identification in a gravitaxis screen [33] and is predicted computationally and suggested by EST data to produce four different protein isoforms with distinct N-terminal domains that share a mostly common C-terminal region bearing two highly conserved, tandemly arranged RNA recognition motifs (RRMs; Figure 1A). Isoforms B/D and E contain an additional 10 amino acid linker between the RRM domains, and all isoforms except B/D contain a 7 amino acid stretch at the C-terminus. Unlike core *gypsy* insulator proteins, Shep is conserved between flies and vertebrates (data not shown).

We confirmed the Mod(mdg4)2.2-Shep physical interaction *in vitro* using recombinant proteins. GST-fusions of Shep isoforms A, B/D, and E (Figure 1B–1C, lanes 4–6) in comparison to GST-Su(Hw) as a positive control (lane 3) and GST alone as a negative control (lane 2) were isolated from bacterial extracts and tested for their ability to interact with purified recombinant His-Mod(mdg4)2.2. His-Mod(mdg4)2.2 is detected in the bound fraction in association with Su(Hw) and each Shep isoform but not GST alone, indicating a direct protein-protein interaction between Shep and Mod(mdg4)2.2.

Similarly, we found that Shep also can interact directly with Su(Hw). GST-fusions of Shep isoforms A, B/D, and E (Figure 1D–1E, lanes 4–6) in comparison to GST alone (lane 2) and positive control, GST-Mod(mdg4)2.2 (lane 3), were tested for their ability to interact with purified recombinant His-Su(Hw). His-Su(Hw) is detected in the bound fraction in association with Mod(mdg4)2.2 and each Shep isoform but not GST alone. For both Su(Hw) and Mod(mdg4)2.2 binding assays, a near 1∶1 molar binding ratio between insulator proteins and Shep was observed, similar to the ratios observed between Mod(mdg4)2.2 and Su(Hw) in both binding experiments. These data provide evidence for direct protein interaction between Shep and Mod(mdg4)2.2 as well as between Shep and Su(Hw).

### Coimmunoprecipitation of *gypsy* insulator proteins with Shep isoforms

**Figure 2:**
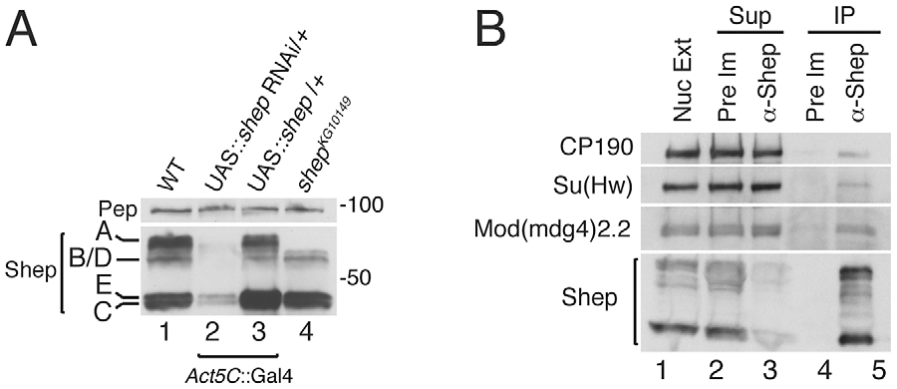
Coimmunoprecipitation of *gypsy* insulator proteins with Shep isoforms. (A) Identification of Shep isoforms *in vivo*. Western blotting for Shep from larval extracts that are wildtype (lane 1), expressing *Act5C*::Gal4 driving single copy UAS-*shep* dsRNA (lane 2), expressing *Act5C*::Gal4 driving single copy UAS-*shep* C and E (lane 3), or containing a P-element insertion that disrupts the coding region of isoform A (lane 4). Pep is shown as a loading control. (B) Coimmunoprecipitation of *gypsy* insulator proteins with Shep. Embryo nuclear extracts (lane 1) were immunoprecipitated (IP) with either Pre-Immune (Pre Im; lanes 2 and 4) or α-Shep (lanes 3 and 5) serum. Shep, Mod(mdg4)2.2, Su(Hw), and CP190 were detected in nuclear extracts (Nuc Ext), supernatants (Sup) (lanes 2–3) and IPs (lanes 4–5) by Western blotting. Approximately 0.02% CP190, 0.02% Su(Hw), and 0.1% Mod(mdg4)2.2 of total were recovered in the IP.

Shep polyclonal antisera were generated using the common C-terminal region downstream of the RRMs allowing detection of all isoforms. Multiple bands are detected in larval extracts by Western blotting, and isoforms A, B/D, C and E were inferred by predicted molecular weights of 68, 60, 45, and 44 kDa, respectively (Figure 2A, lane 1). All bands are depleted upon *shep* RNAi hairpin knockdown, which targets all isoforms (lane 2), indicating antibody specificity. When the *shep^EY04794^* allele, which contains a UAS sequence upstream of the *shep* C and E promoter, is induced ubiquitously using Gal4, the 45 kDa doublet is enriched over wildtype, identifying these two bands as isoforms C and E (lane 3). Finally, homozygous P-element insertion in *shep^KG10149^* predicted to disrupt translation of isoform A causes specific loss of the largest band (lane 4). By process of elimination, isoform B/D corresponds to the apparent 60 kDa band.

We used our specific Shep antisera to test whether *gypsy* insulator proteins associate with Shep *in vivo* by coimmunoprecipitation. When Shep complexes are immunoprecipitated from embryo nuclear extracts using Shep or control preimmune antisera, Shep is efficiently purified with the specific antibody (Figure 2B). Furthermore, a fraction of total *gypsy* insulator proteins CP190, Su(Hw) and Mod(mdg4)2.2 are detected in the bound fraction in association with Shep. The Polycomb Group (PcG) proteins, Pc and E(z) are not purified in the bound fraction, indicating specificity of the interaction between Shep and insulator proteins (Figure S1). Therefore, these data demonstrate that Shep interacts by direct protein interactions with Mod(mdg4)2.2 and Su(Hw) *in vitro* and associates with *gypsy* insulator proteins *in vivo*.

### Identification of *shep* loss-of-function alleles

**Figure 3:**
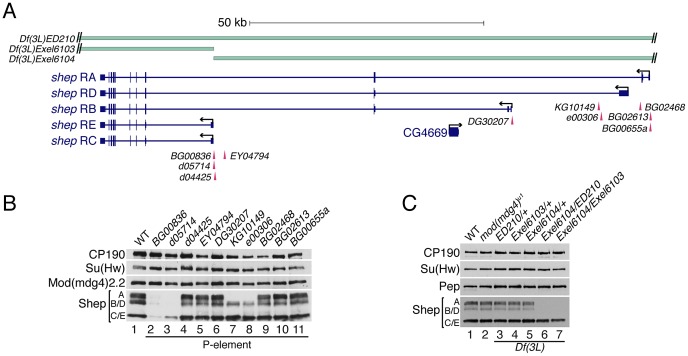
Identification of Shep loss-of-function alleles. (A) Diagram of lesions in the *shep* locus. P-element insertion sites are denoted below the gene model, and genomic deficiencies are indicated above the gene model. Hatched lines indicate that deletions extend beyond the *shep* locus. See Table 1 for P-element details. (B) Western blotting of larval extracts of *mod(mdg4)* ^+^ and homozygous *shep* P-element insertion larval extracts for Shep, Mod(mdg4)2.2, Su(Hw), and CP190 in the *mod(mdg4)* ^+^ background. Lane numbers of gel are indicated. (C) Western blotting for CP190, Su(Hw), Pep, and Shep in larval extracts of *mod(mdg4)* ^+^, *mod(mdg4)^u1^*, and heterozygous or transheterozygous *shep* deficiencies in the *mod(mdg4)^u1^* background.

Direct physical interaction between Shep and *gypsy* insulator proteins prompted us to examine the functional relationship between *shep* and the *gypsy* insulator. We first obtained and characterized *shep* alleles bearing either *P*-element insertions or FRT-derived deletions independently generated from seven different genetic backgrounds [Figure 3A]; [ Table 1; 34,35]. To determine whether these alleles are loss-of-function, we performed quantitative RT-PCR for total and specific *shep* isoform transcript levels and observed decreases in larvae hemizygous for *shep* or containing homozygous *shep* P-element insertions (data not shown). Furthermore, four different homozygous P-element insertions result in loss of Shep protein, two greatly reducing all isoforms (Figure 3B, lanes 2–3) and two eliminating isoform A (lanes 7–8). No changes in Shep protein were observed when P-element alleles are heterozygous (data not shown), suggesting that these mutations are recessive. Additionally, *Df(3L)Exel6104* transheterozygous deficiency combinations are viable and retain isoforms C and E, suggesting that isoforms A and B/D are not essential (Figure 3C, lanes 6–7). Other transheterozygous combinations of deficiencies or homozygous deficiencies cause lethality (Table 1), but due to deletion of neighboring essential genes, we cannot determine whether *shep* itself is essential for viability using these alleles. Importantly, no change in CP190, Su(Hw) or Mod(mdg4)2.2 protein levels is observed in *shep* mutants relative to wildtype levels (Figure 3B–3C). These data show that P-element insertions and deficiencies decrease Shep protein levels and likely constitute loss-of-function alleles.

**Table 1:** Summary of *shep* homozygous P element and heterozygous deficiency alleles.

Name	Protein Isoform(s) Disrupted	Synthetic Lethality^1^	Effect on *ct6* phenotype^1^	Viability crossed to *Df(3L)ED 210* 1	Effect on *ct6*with *Df(3L)ED 210* 1	Viability crossed to *Df(3L)Exe l6103* 1	Effect on *ct6*with *Df(3L)Exe l6103* 1	Viability crossed to *Df(3L)Exe l6104* 1	Effect on *ct6*with *Df(3L)Exe l6104* 1	P element type	Markers	Deletion Span
*BG00836*	All	Strong (9.2%)	None^4^	Synthetic Lethality	Positive^4^	Synthetic Lethality	Positive^4^	Viable^2^	Positive^2^	GT1	*w+*	-
*d05714*	All	Strong (23%)	Positive^4^	Synthetic Lethality	Positive^4^	Synthetic Lethality	Positive^4^	Viable^2^	Positive^2^	XP	*w+*	-
*d04425*	None	None (103%)	Positive	Viable	Positive	Viable	Positive	Viable	Positive	XP	*w+*	-
*EY04794*	None	NQ	None	Viable	Positive	Viable	Positive	Viable	Positive	EY	*w+*, *y+*	-
*DG30207*	None	NQ	Positive	Viable	Positive	Viable	Positive	Viable	Positive	wHy	*w+*, *y+*	-
*KG10149*	A	Weak (63%)	Positive	Viable	Positive	Viable	Positive	Viable	Positive	SUPor-P	*w+*, *y+*	-
*e00306*	A	Weak (79%)	Positive	Viable	Positive	Viable	Positive	Viable	Positive	RB	*w+*	-
*BG02468*	None	NQ	Positive	Viable	Positive	Viable	Positive	Viable	Positive	GT1	*w+*	-
*BG02613*	None	Weak (57%)	Positive	Viable	Positive	Viable	Positive	Viable	Positive	GT1	*w+*	-
*BG00655a*	None	Weak (64%)	Positive	Viable	Positive	Viable	Positive	Viable	Positive	GT1	*w+*	-
*Df(3L)ED210*	All	NR^3^	Positive^5^	NR^3^	NR^3^	NR^3^	NR^3^	Viable^2^	Positive^2^	-	*w+*	3L:4,544,2 34 -3L:5,348,4 42
*Df(3L)Exel6103*	All	NR^3^	Positive^5^	NR^3^	NR^3^	NR^3^	NR^3^	Viable^2^	Positive^2^	-	*w+*	3L:4,976,4 03 -3L:5,177,8 96
*Df(3L)Exel6104*	A, B, D	NR^3^	Positive^5^	Viable	Positive	NR^3^	Positive	NR^3^	NR^3^	-	*w+*	3L:5,177,8 96 -3L:5,359,1 62

1 In *mod(mdg4)^u1^* background; percentage shown is % viable homozygous adults with respect to number of homozygous' pupae; NQ = not quantified; see Table S2 for number of flies and pupae counted.

2 Expresses isoforms C and E.

3 No results due to lethality.

4 Escaper phenotype.

5 Scored as heterozygotes.

### Synthetic lethal relationships between *mod(mdg4)* and *shep*

We observed that *mod(mdg4)* mutants are particularly sensitive to *shep* expression levels. Homozygous *shep* P-element insertion alleles are viable in a wildtype background; however, in combination with *mod(mdg4)^u1^*, which is fully viable but null for the *mod(mdg4)2.2* isoform, homozygous *shep* mutants displaying reduced Shep protein specifically exhibit strongly reduced viability (Table 1). We observed lethality in late pupal development and pharate adults; only 9.2% of *shep^BG00836^* and 23% of *shep^d05714^ mod(mdg4)^u1^* double mutant pupae survive to adulthood. Synthetic lethality was also observed for *shep* mutant alleles in combination with the *mod(mdg4)^T6^* loss-of-function point mutation, confirming the genetic interaction. Moreover, overexpression of the *shep^EY04794^* allele containing a UAS insertion or the Shep E isoform from a transgenic copy inserted on a different chromosome using the *Act5C*::Gal4 driver causes complete inviability of adult flies in the *mod(mdg4)^u1^* background but not in wild type. In contrast, overexpression of the Shep E isoform harboring point mutations in the RRM domain designed to disrupt RNA-binding activity but not protein folding does not cause lethality in *mod(mdg4)^u1^* flies despite both versions of Shep E protein being expressed at the same levels in wildtype flies (data not shown). The apparent sensitivity of *mod(mdg4)^u1^* null mutants to alterations in Shep levels is consistent with direct physical interactions between Shep and insulator proteins and further suggests an antagonistic functional relationship between Mod(mdg4)2.2 and Shep, likely requiring Shep RNA-binding activity.

### Shep negatively regulates *gypsy* enhancer blocking activity

**Figure 4:**
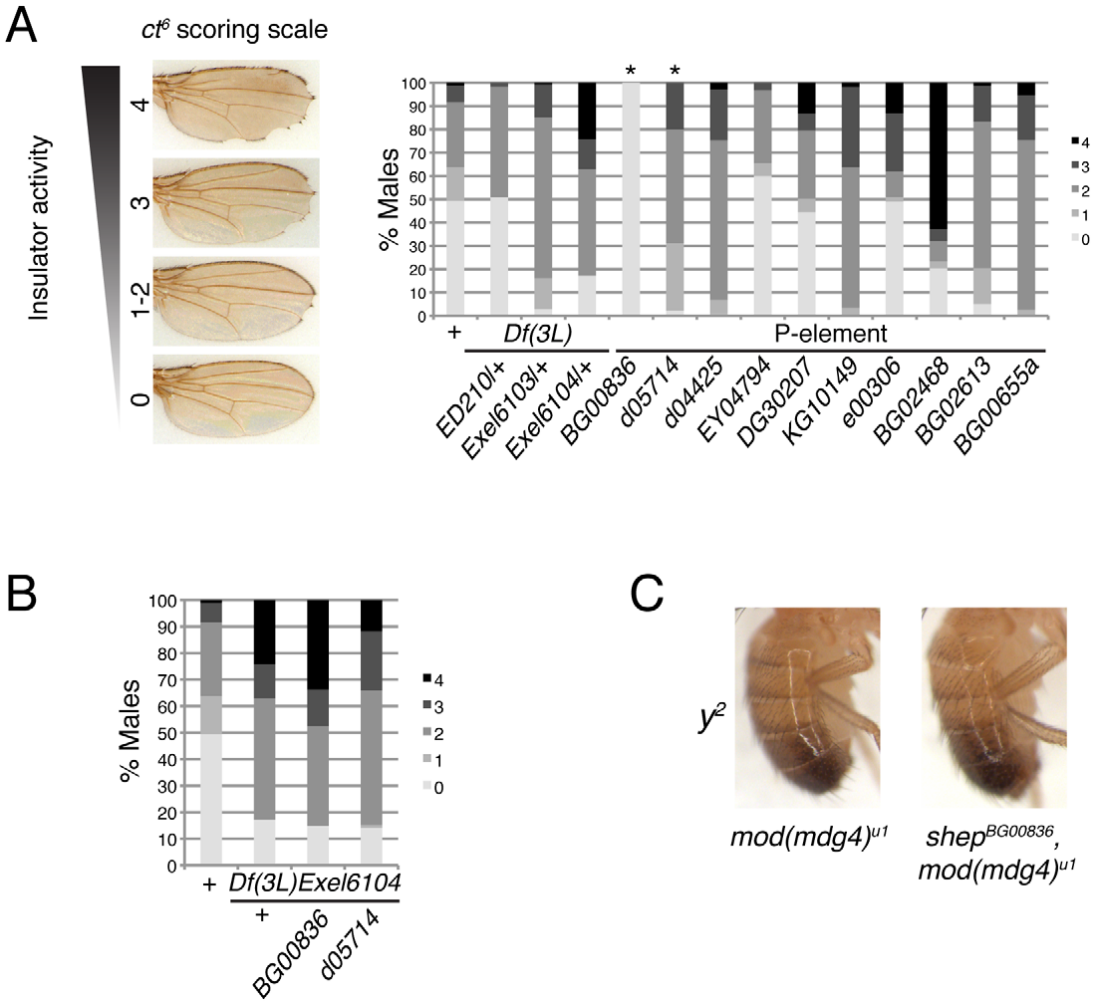
Loss-of-function *shep* alleles disrupt *gypsy* insulator activity at *ct^6^*. (A) Effects of *shep* mutations on the *ct^6^* phenotype. All flies are homozygous for *mod(mdg4)^u1^*. At the *shep* locus, flies are wildtype (*shep^+^*), harbor a heterozygous deficiency, or contain a homozygous P-element insertion as indicated. Percent of population scored on a scale of 0–4 is indicated for each genotype. 0, no notching; 1, slight notching in one wing; 2, slight notching in both wings; 3, pronounced notching in hinge distal wing margin; 4, severe notching in both hinge proximal and distal margins. Asterisks denote P-element insertions showing extensive synthetic lethal interaction with *mod(mdg4)^u1^* for which rare escapers were scored (49≤*n*≤180 for all genotypes). (B) Hemizygous alleles of *shep* affect *ct^6^*. Phenotypes of *ct^6^* of *shep^BG00836^* and *shep^d05714^* mutations transheterozygous with *Df(3L)Exel6104*. All flies are homozygous for *mod(mdg4)^u1^*. Flies were scored in parallel with those in (A) (85≤*n*≤180). (C) Male abdominal pigmentation due to *y^2^* expression is unchanged in *mod(mdg4)^u1^* compared to *shep^BG00836^*, *mod(mdg4)^u1^* flies.

In order to assess whether *shep* loss-of-function affects insulator activity *in vivo*, we examined the phenotypes of two well-characterized *gypsy*-dependent alleles, *y^2^* and *ct^6^*. These alleles result from *gypsy* retrotransposon insertion between the upstream body enhancer and promoter of *y* or between the upstream distal wing margin enhancer and promoter of *ct* [36]. These insertions block enhancer function, resulting in loss of abdominal cuticle pigmentation or misshapen wing margin, respectively. In an otherwise wildtype background, *shep* P-element alleles and deficiencies produce no decrease in enhancer blocking activity at *y^2^* or *ct^6^* (data not shown), and since *y^2^* and *ct^6^* are fully active for enhancer blocking, an increase in insulator activity cannot be assessed. In order to sensitize the assay, *y^2^* and *ct^6^* were examined in the presence of the *mod(mdg4)^u1^* mutation. This mutation disrupts insulator function and allows partial restoration of enhancer-promoter communication. The *gypsy*-dependent phenotypes in homozygous *shep* P-element alleles in the *mod(mdg4)^u1^* background were scored for *ct^6^* on a scale of 0–4 with increasing severity of phenotype. Approximately half of male *mod(mdg4)^u1^* wings display a score of zero (Figure 4A). In contrast, for eight of ten homozygous P-element and all heterozygous deficiency alleles of *shep*, we observed positive effects on enhancer blocking activity at *ct^6^* in the *mod(mdg4)^u1^* background (Figure 4A, Table 1, Table S1), indicating increased *gypsy* insulator activity. For *shep^BG00836^* and *shep^d05714^ mod(mdg4)^u1^* double homozygous mutants, only escapers could be scored due to synthetic lethality. Similar changes in insulator phenotypes were observed for *shep* hemizygous mutations (Figure 4B) but not heterozygous mutations in the *mod(mdg4)^u1^* background (data not shown), indicating that these *shep* mutations are recessive with respect to insulator activity.

To verify that the P-element insertion alleles are loss-of-function for enhancer blocking activity, the insulator phenotypes of each *shep* P-element allele crossed to each deficiency were examined. We found that insulator phenotypes and synthetic lethality remained the same or insulator function was slightly increased compared to homozygous P-elements, except when *shep^BG00836^* and *shep^d05714^* are transheterozygous with *Df(3L)Exel6104* (Table 1). In these cases, synthetic lethality is rescued, corresponding to elevated isoform C and E transcript and protein levels likely due to artificial juxtaposition of the C and E promoter to a *cis*-regulatory element from a partially deleted upstream gene or mini-*w^+^*of the original P-element remaining after FRT excision (data not shown). Nevertheless, insulator activity of these *shep^BG00836^* and *shep^d05714^* transheterozygous mutants is improved compared to *mod(mdg4)^u1^*, confirming that *shep^BG00836^* and *shep^d05714^* are loss-of-function alleles (Figure 4B).

We determined that *shep* P-element mutants in the *mod(mdg4)^u1^* background do not affect the phenotype of *ct^n^*, caused by insertion of a *roo* transposable element (data not shown). This result suggests that the effect of *shep* on *ct^6^* is due to changes in *gypsy* insulator activity and not direct regulation of *ct* expression. Importantly, since *shep* mutants affect insulator activity in *mod(mdg4)^u1^* null mutants, it likely that, *in vivo*, Shep can interact with Su(Hw) in the absence of Mod(mdg4)2.2. Overall, these data indicate that the wildtype function of Shep is to negatively regulate *gypsy* insulator activity.

In contrast to positive effects on *ct^6^*, *shep* mutations in the *mod(mdg4)^u1^* background do not affect *y^2^*. The phenotype of *y^2^* remained unchanged by mutation or deletion of *shep* in the *mod(mdg4)^u1^* background (Figure 4C, data not shown). The specific effect at *ct^6^* but not *y^2^* in *shep* mutants raises the possibility that *shep* negatively regulates a subset of *gypsy* insulators.

### Shep alters *gypsy* insulator localization in a tissue-specific manner

**Figure 5:**
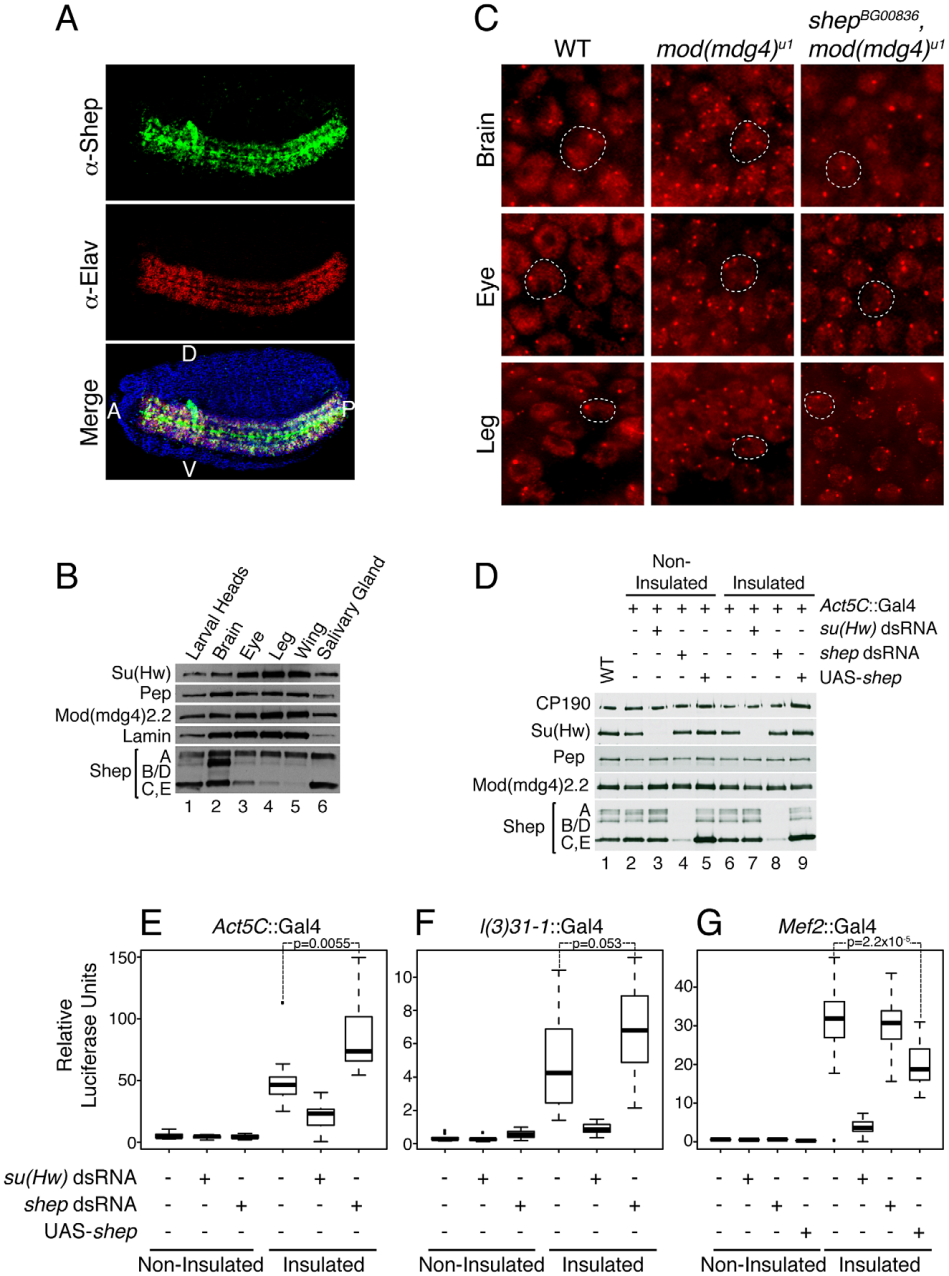
Shep negatively regulates *gypsy* activity in the CNS. (A) Confocal imaging of Shep distribution in stage 14 wildtype Oregon R embryo by indirect immunofluorescence using guinea pig α-Shep (green) and mouse α-Elav (red) antibodies detected by α-guinea pig Alexa-488 and α-mouse Alexa-594 secondary antibodies. DAPI staining (blue) is also shown in the merged image. A, anterior; P, posterior; D, dorsal; V, ventral. (B) Western blotting of anterior third instar larval extracts (lane 1), brains (lane 2), eye discs (lane 3), leg discs (lane 4), wing discs (lane 5), and salivary glands (lane 6) for Shep, Su(Hw), Mod(mdg4)2.2, Pep, and Lamin. (C) Epifluorescence imaging of insulator body localization by indirect immunofluorescence using rabbit α-CP190 and α-rabbit Alexa-594 in whole mount brain, leg imaginal disc, or eye imaginal disc tissues in wild type; *mod(mdg4)^u1^*; or *shep^BG00836^*, *mod(mdg4)^u1^* larvae. White dotted lines outline one example nucleus in each image. (D) Western blotting of larval extracts for Shep, Su(Hw), CP190, Mod(mdg4)2.2 and Pep in wildtype (lane 1), non-insulated (lanes 2–5), and insulated (lanes 6–9) luciferase lines. *Act5c*::Gal4 was used to drive single copy UAS-*su(Hw)* dsRNA (lanes 3 and 7), UAS-*shep* dsRNA (lanes 4 and 8) or Shep overexpression (UAS-*shep*, lanes 5 and 9). (E–G) Relative luciferase units were quantified in individual larvae expressing *Act5C*::Gal4 (E), *l(3)31-1*::Gal4 (F) *Mef2*::Gal4 (G), dsRNA hairpin, and/or UAS-*shep* as indicated. Luciferase values across the population are plotted as box and whisker plots where boxes represent upper and lower quartiles proximal to the median, and whiskers represent the range excluding outliers. Populations were compared by 1-way ANOVA, and pairwise *p* values were calculated by Tukey HSD *post hoc* tests. Outliers falling outside a normal distribution are shown (dots) but were not used to calculate *p* values. For each genotype, *n*≥12 larvae. For (F), non-insulated control vs. non-insulated *shep* RNAi, *p* = 0.18; for (G), insulated control vs. insulated *shep* RNAi, *p* = 0.99.

In order to determine how Shep regulates insulator function and in what contexts, we examined the distribution of Shep in late stage wildtype embryos. We find that Shep protein is enriched in the embryonic CNS including the brain and ventral nerve cord, areas that are also positive for the neuron-specific protein Elav (Figure 5A). The overlap between Shep and Elav is partial in that Shep is also expressed in glial cells. Shep levels are low but detectable in non-CNS tissues; likewise, microarray expression data from various developmental stages are consistent with our results [37]. In the third instar larval stage, higher overall protein levels are detected in the brain compared to eye, leg, or wing imaginal discs or salivary glands by Western blotting (Figure 5B) as well as immunofluorescence (data not shown). These data demonstrate that Shep is a CNS-enriched protein at both embryonic and larval stages.

In order to examine whether Shep affects insulator complexes in a tissue-specific manner, we examined the localization of insulator bodies in the presence and absence of Shep in larval brain compared to non-CNS cell types. Wild type, *mod(mdg4)^u1^* and double mutant *shep^BG00836^*, *mod(mdg4)^u1^* whole mount larval brain and imaginal disc tissues were stained using antibodies directed against CP190. Because the brain contains heterogeneous cell types, we focused on peripheral cells in the medulla of the brain lobe in which 1–2 insulator bodies are visible in the nucleus per focal plane. In *mod(mdg4)^u1^* mutants, insulator bodies are disrupted in all tissues including the brain, resulting in an increased number of foci compared to wild type (Figure 5C). In *shep^BG00836^*, *mod(mdg4)^u1^* double mutants, insulator body localization in the brain reverts to a wildtype appearance (observed in 8 of 9 experiments). The same effect is also observed in perineurial glia of the outer cell layer surrounding the brain hemispheres (data not shown). In contrast, peripheral cells of the eye and leg imaginal discs, which display low Shep expression, insulator bodies are indistinguishable in *shep^BG00836^*, *mod(mdg4)^u1^* compared to *mod(mdg4)^u1^* mutants. We also did not observe differences in CP190 localization in peripheral cells of the wing imaginal disc; however, insulator bodies in all genotypes are less prominent in this tissue type (data not shown). Additional *shep* mutants examined, *shep^KG10149^*, *shep^e00306^*, *shep^BG00655a^*, and *shep^BG02613^*, display similar effects (data not shown). Restoration of mislocalized insulator bodies when *shep* levels are reduced in the brain but not non-CNS tissue suggests a tissue-specific role for Shep in disrupting insulator activity.

### Shep represses insulator barrier activity in CNS tissue

In order to determine whether Shep affects insulator activity in the CNS, we developed a versatile barrier assay that allows quantification of *gypsy* insulator activity using identical reporters in essentially any tissue of interest. This assay relies on three transgenes: the transcriptional reporter UAS-luciferase inserted into a defined *attP* landing site, either insulated by flanking Su(Hw) binding sites or non-insulated [38]; a Gal4-inducible dsRNA hairpin construct for knockdown of a gene of interest [39]; and a tissue-specific Gal4 driver. This system allows for directly comparable quantification of luciferase activity in the insulated or non-insulated context in the presence or absence of a protein of interest. Use of the Gal4 system allows interrogation of a specific subset of cells for both the reporter as well as the hairpin knockdown within an otherwise wildtype organism, which is not easily achieved using standard genetic manipulation of existing mutants. We used luciferase reporter constructs inserted into *attP3* on the X chromosome [40], which display extremely low basal expression unless insulated (Figure 5E–5G) relative to other *attP* insertion sites tested [38]. Insulator-dependent expression at *attP3* is likely due to its positioning within a PcG repressed region (Figure S2). Addition of insulators flanking the UAS-luciferase reporter likely stops the spread of repressive chromatin, allowing for measurable activity. Due to high variability of expression among individuals, luciferase levels were measured in individual whole third instar larvae, and values for each population (*n*≥12) were compared by one-way ANOVA. As proof of principle, ubiquitously expressed *Act5C*::Gal4 induces high luciferase activity in insulated compared to non-insulated lines (Figure 5E). As expected, *su(Hw)* knockdown causes a drastic reduction in both Su(Hw) protein (Figure 5D, lanes 3 and 7) and luciferase activity in insulated but not non-insulated lines (Figure 5E), indicating that luciferase expression directly reports Su(Hw)-mediated insulation. In contrast, upon *shep* knockdown (Figure 5D, lanes 4 and 8) an increase in luciferase activity is observed for the insulated line (*p* = 0.0055, Tukey's HSD *post hoc* test), indicating an increase in insulator activity (Figure 5E). Therefore, Shep negatively influences both *gypsy-*dependent barrier and enhancer blocking activities.

Since ubiquitous knockdown of *shep* could report an increase in insulator activity in any or all tissues, CNS-specific Gal4 expression was utilized to quantitatively address whether *shep* affects *gypsy* insulator activity in the CNS. Localized Gal4 expression in the CNS with *l(3)31-1*::Gal4 induces luciferase to a lower level than ubiquitous Gal4 due to its restricted expression pattern (Figure 5F). Upon *su(Hw)* knockdown in the CNS, luciferase expression returns to non-insulated levels. In contrast, when *shep* is knocked down, a marginally significant increase in luciferase levels is observed (*p* = 0.053), demonstrating that *shep* negatively affects insulator activity in the CNS.

Finally, we tested whether Shep affects barrier activity in muscle cells, a tissue type that expresses low levels of Shep. Muscle-specific *Mef2*::Gal4 induces high levels of luciferase activity; accordingly, *su(Hw)* knockdown results in a dramatic decrease in luciferase activity (Figure 5G). In contrast, *shep* knockdown in muscle tissue has no significant effect compared to *Mef2*::Gal4 alone (*p* = 0.99), demonstrating that *shep* does not play a substantial role in insulator activity in muscle tissue. However, ectopic overexpression of Shep C and E using *shep^EY04794^* in muscle tissue is sufficient to result in decreased insulator activity (*p* = 2.2×10^−5^). Therefore, in muscle cells, artificially reaching a certain threshold of Shep protein expression reduces insulator activity. This quantitative and tissue-specific insulator assay further supports a role for Shep as a negative regulator of *gypsy* insulator activity.

### Comparison of Shep and *gypsy* insulator protein genome-wide localization

**Figure 6:**
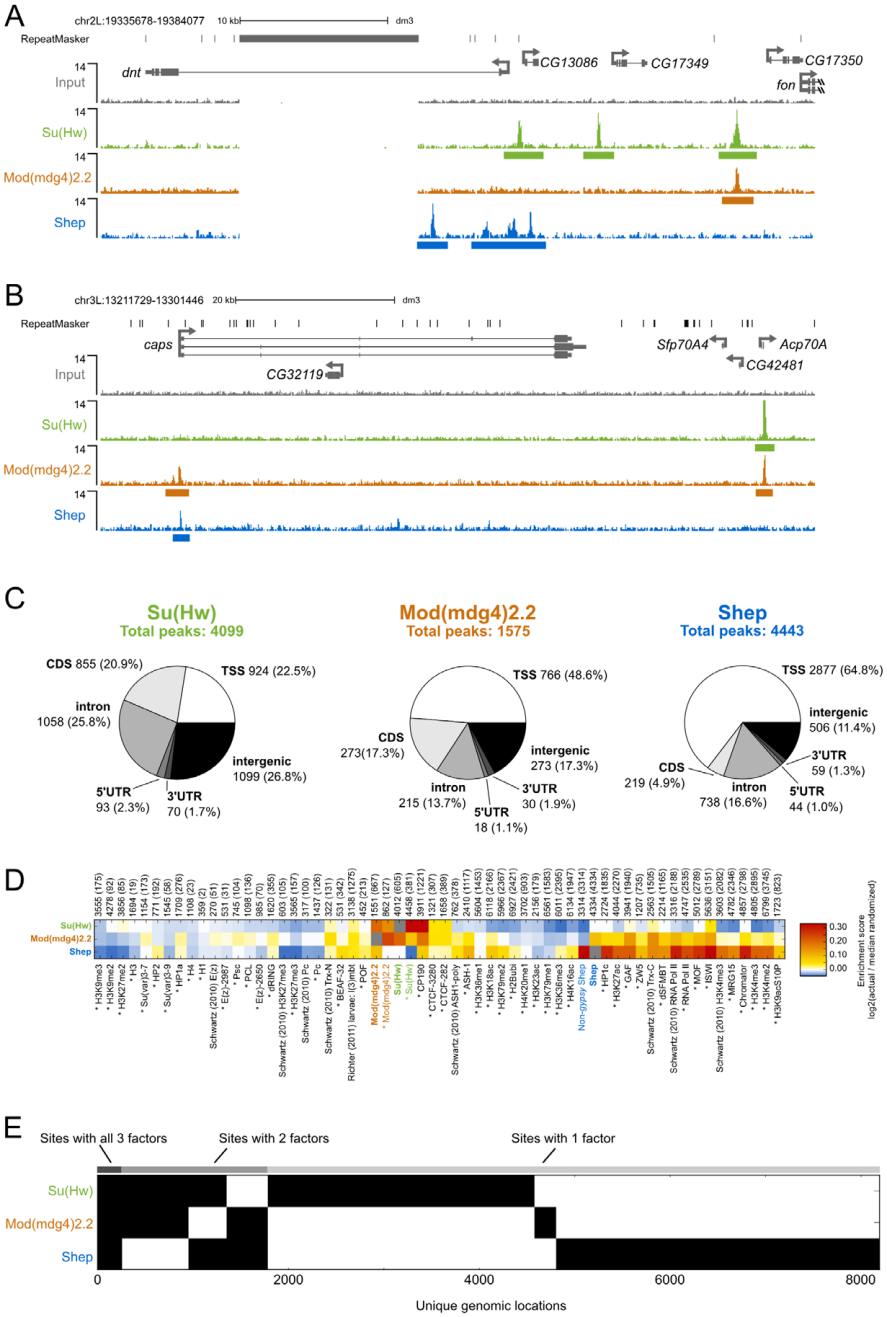
Comparison of Su(Hw), Mod(mdg4)2.2, and Shep ChIP–seq profiles in BG3 cells. (A) Screenshot of Su(Hw), Mod(mdg4)2.2, and Shep ChIP-seq signals at the *dnt* neuronal-expressed locus. The large gap in ChIP signal corresponds to a highly repetitive region to which sequence reads could not be aligned with high confidence. (B) Screenshot of the *caps* neuronal-expressed locus. (C) Classification of Su(Hw), Mod(mdg4)2.2, and Shep ChIP-seq peaks in BG3 cells. Number of sites and percentage of total in parentheses corresponding to TSS, transcription start site; CDS, coding sequence; 5′ UTR, 5′ untranslated region; 3′ UTR, 3′ untranslated region. See methods for classification hierarchy of overlapping categories. (D) Heat map of log_2_ enrichment scores for pairwise comparisons of binding sites for Su(Hw), Mod(mdg4)2.2, Shep, and additional data sets. Color scale corresponding to enrichment value is indicated (right). Positive values indicate significant enrichment while negative values indicate significant negative correlation of enrichment. Self-self comparisons are indicated in grey, and pairwise comparisons that are not statistically significant (p>0.001) are indicated in white. Numbers along top of each column indicate the total number of features in each data set, and the number of sites overlapping with Shep are indicated in parentheses. Data from Richter (2011) were derived from larval brains and imaginal discs; all other datasets are derived from BG3 cells. Data from modENCODE are indicated by an asterisk. Full heat map with hierarchical clustering is shown in Figure S4. (E) Binary heat map of Su(Hw), Mod(mdg4)2.2, and Shep binding sites in BG3 cells ordered by supervised hierarchical clustering. Each row represents a single genomic location, and a mark in a column represents the presence of a particular factor.

In order to determine the extent to which Shep colocalizes with insulator proteins, we mapped the genome-wide chromatin association profiles of Su(Hw), Mod(mdg4)2.2, and Shep by ChIP-seq in the BG3 larval CNS-derived cell line. Using previously characterized Su(Hw) and Mod(mdg4)2.2 antibodies [16], [41], [42] as well as our specific Shep antisera (see methods), we observe sharp peaks of Su(Hw), Mod(mdg4)2.2, and Shep, as well as broader peaks of Shep signal (Figure 6A–6B). Using the SPP algorithm [43] at a 1% false discovery rate (FDR), we detected 4099 Su(Hw) peaks, 1575 Mod(mdg4)2.2 peaks, and 4443 Shep peaks (Figure 6C), numbers in agreement with previous studies of Su(Hw) and Mod(mdg4)2.2 binding profiles in various cell types [9], [10], [42]. Similar to previous studies [10], [42], [44], the majority of Mod(mdg4)2.2 sites overlap with Su(Hw), and strong enrichment of overlap is observed compared to random expectation (Figure 6D). As expected, Su(Hw) is found mostly in inter- and intragenic regions [9], [10], [44] (Figure 6C). In contrast, Shep binding is mainly observed over genes, with 65% of Shep peaks falling in transcription start sites (TSSs). An intermediate distribution pattern is observed for Mod(mdg4)2.2.

Given that Shep can interact directly with either Su(Hw) or Mod(mdg4)2.2 and copurifies with a fraction of total *gypsy* insulator core proteins, we expected a substantial degree of overlap between Shep and either Su(Hw) or Mod(mdg4)2.2. Indeed, nearly half of Mod(mdg4)2.2 sites overlap with Shep, and 16% of Shep sites overlap with Mod(mdg4)2.2 (Figure 6E). The observed overlap between Shep and Mod(mdg4)2.2 is greater than random expectation (Figure 6D). In contrast, no enrichment is observed for colocalization between Shep and Su(Hw). Nevertheless, nearly one quarter of Shep binding sites overlap with either Su(Hw) or Mod(mdg4)2.2 (Figure 6E), supporting the notion that a substantial fraction of chromatin-associated Shep harbors insulator-related activity. Although expressed at low levels in salivary glands, Shep localization in polytene chromosomes also shows partial overlap between Shep and *gypsy* insulator proteins (Figure S3). Chromatin association of Shep at non-*gypsy* insulator sites could reflect alternate unknown functions of Shep or a *gypsy* insulator-independent means of recruitment.

We next compared Shep genome-wide localization with that of a variety of chromatin-associated factors and histone modification marks in BG3 cells. Enrichment scores for two-way overlaps between all factors were calculated, and unsupervised hierarchical clustering was performed (Figure S4). This analysis reveals high similarity of binding profiles of the insulator proteins Su(Hw), Mod(mdg4)2.2, CP190, and CTCF (Figure 6D). In contrast, Shep genome-wide localization most closely resembles factors associated with active transcription such as RNA polymerase II. Analysis of Shep sites not overlapping with either Su(Hw) or Mod(mdg4)2.2 also overlap significantly with active transcription marks. Consistent with our comparative analysis, Shep localization is likewise observed at highly transcribed puff regions of polytene chromosomes (Figure S3). Interestingly, Shep genome-wide localization also displays similarity to that of Chromator, a protein recently implicated as a boundary factor potentially capable of organizing physical chromatin domains [25] and also overlaps significantly with CP190 and BEAF (Figure 6D).

Consistent with Shep functioning as a negative regulator of *gypsy* insulator activity, we noted a significantly lower than expected frequency of three-way overlap among Shep, Su(Hw) and Mod(mdg4)2.2. In fact, the three factors are only observed together at 271 sites (Figure 6E). Considering the 1403 Mod(mdg4)2.2 sites that colocalize with either Su(Hw) or Shep, this degree of three-way overlap is lower than expected by chance (*p*<1×10^−4^, permutation test; *p* = 2.2×10^−16^, hypergeometric test). The same results are obtained when this analysis is performed on Su(Hw) sites that overlap with either Mod(mdg4)2.2 and Shep as well as the Shep sites that overlap with either Su(Hw) or Mod(mdg4)2.2 (see methods). Taken together, these results indicate substantial colocalization of Shep with Mod(mdg4)2.2 but limited three-way overlap among Shep and both *gypsy* insulator proteins.

## Discussion

Here we have demonstrated a role for the CNS-enriched RRM protein, Shep, in the tissue-specific, negative regulation of *gypsy* chromatin insulator activity. Shep interacts directly with either Su(Hw) or Mod(mdg4)2.2 *in vitro* and associates physically with *gypsy* insulator complexes *in vivo*. Mutations in *shep* improve enhancer blocking activity and cause synthetic lethality with *mod(mdg4)2.2* mutations. Two lines of evidence indicate that Shep affects insulator activity in a tissue-specific manner. First, insulator body localization is altered in CNS but not other tissues of *shep* mutants. Second, barrier activity is improved in CNS but not muscle tissue when Shep levels are reduced. Finally, genome-wide mapping of Shep and *gypsy* insulator proteins in BG3 cells reveals substantial overlap with individual insulator proteins but lack of three-way overlap, further supporting a role for Shep in negative regulation of insulator activity in certain tissues.

### Shep negatively regulates *gypsy* insulator activity in a tissue-specific manner

Shep acts as a tissue-specific negative regulator of *gypsy* insulator function and insulator body localization. Shep localization is most enriched in the CNS at both embryonic and larval stages; however, it is also expressed at lower levels in additional tissues. Although we have demonstrated that Shep functions in the CNS, Shep can also repress enhancer blocking activity in the wing and could possibly affect insulator activity in other tissues. For example, ubiquitous reduction of Shep levels strongly improves overall barrier activity, suggesting that tissues outside of the CNS may also harbor Shep activity. Nonetheless, Shep does not appear to function in all tissues; knockdown of Shep does not affect barrier activity in muscle tissue, no changes in insulator body localization are observed in eye or leg tissue of *shep* mutants, and no effect is observed for *y^2^* enhancer blocking in pigment cells of *shep* mutants. Interestingly, when Shep is overexpressed in muscle tissue, reduction of barrier activity is observed, suggesting that a certain threshold of Shep protein is needed to repress insulator activity. Since Shep protein can be detected at least at low levels in all tissues tested thus far, it is unlikely that the mere presence of Shep protein is sufficient to disrupt *gypsy* insulator activity. It remains to be determined what other cofactors, such as proteins or RNAs, may contribute to Shep activity.

Shep may negatively regulate insulator activity by interfering with insulator protein interactions required for their activity. ChIP-seq analyses shows that the genome-wide binding profile of Shep in CNS-derived BG3 cells overlaps substantially with that of Mod(mdg4)2.2 but not extensively with both Su(Hw) and Mod(mdg4)2.2 combined. Lack of three-way overlap is not entirely unexpected given that Shep is a negative regulator of *gypsy* insulator activities. Shep coimmunoprecipitation experiments copurify only a small fraction of total insulator proteins present in nuclear extracts, suggesting that Shep-insulator complexes are not abundant or not stable *in vivo*. Since Shep can bind either Mod(mdg4)2.2 or Su(Hw) *in vitro* at a 1∶1 ratio, Shep binding could compete with direct interaction between Mod(mdg4)2.2 and Su(Hw) or their interactions with other factors such as CP190. Moreover, our finding that *mod(mdg4)* mutants are highly sensitive to Shep dosage suggests an antagonistic functional relationship between Mod(mdg4)2.2 and Shep. Specifically, Shep may negatively regulate higher order insulator-insulator complex interactions, which appear to be mediated by direct interaction between Mod(mdg4)2.2 and CP190 [4]. Insulator body localization in larval brains of *shep*, *mod(mdg4)^u1^* mutants reverts back to a wildtype pattern compared to compromised *mod(mdg4)^u1^* mutants, perhaps indicating that the normal function of Shep may be to prevent larger insulator complexes from forming in these cell types.

### Functional consequences of Shep activity in the CNS

Our results are consistent with the possibility that Shep promotes tissue-specific chromatin configurations by modulating insulator complexes. While differential occupancy of insulator proteins at their respective binding sites may play a role in regulating certain loci [21], occupancy throughout the genome does not differ extensively between cell types [9], [45]. Therefore, alternate mechanisms to control insulator activity likely exist. Shep activity could prevent insulator-insulator contacts otherwise present in tissues that do not express *shep*, resulting in relief of enhancer blocking or repression by silencers. Interestingly, *shep* was identified as a regulator of complex behavioral traits in screens for altered sensory-motor responsiveness to gravity [33] and aggressive behavior [46], suggesting the possibility that regulation of an insulator-based mechanism could exist to effect changes in neurological function.

### Potential involvement of RNA in Shep and chromatin insulator function

Given that Shep is an RRM-containing protein, RNA-binding may contribute to the ability of Shep to associate with insulator complexes *in vivo*. Shep RRMs are highly conserved, and lethality caused by Shep overexpression in the *mod(mdg4)* mutant background is not observed when the RRMs are mutated. This result suggests that Shep RRMs may be functional with respect to insulator activity. One possibility is that the specific RNA bound by Shep could affect targeting of Shep to insulator sites. Another not mutually exclusive prospect is that Shep is recruited to chromatin cotranscriptionally by binding nascent transcripts. It will be important to determine in future studies if Shep binds RNA while in complex with *gypsy* insulator proteins as well as the identities of Shep and insulator-associated RNA. Our results point to a novel role for Shep and possibly RNA to regulate insulator activity in a tissue-specific manner.

## Materials and Methods

### Drosophila strains

Stocks were raised at 25°C on standard cornmeal medium. *Shep* P-element insertion alleles, *shep* deficiencies, *Act5C*::Gal4, *Mef2*::Gal4, and *l(3)31-1*::Gal4 were obtained from the Bloomington and Exelixis Stock Centers. Lines expressing *su(Hw)* (10724 GD) or *shep* dsRNA (37863 GD) were obtained from the Vienna *Drosophila* RNAi Center. UAS::luciferase constructs were inserted into the *attP3* landing site [38]. The *ct^6^* phenotype was scored in flies on the first day after eclosion. For all genotypes, males show a more severe wing notching phenotype than females. The *y^2^* phenotype was scored in flies aged for 1 d at 25°C. Larvae for luciferase insulator assays and whole mount immunofluorescence were raised at 25°C. Larvae for polytene chromosome staining were raised at 18°C. Embryos aged 0–24 h for nuclear extracts and immunofluorescence were collected from a population cage as described [20]. Anterior thirds of larvae were used for Western blotting.

### Shep cloning

Coding regions of FlyBase annotated Shep isoforms RA, RB/RD, and RE were amplified by PCR from Trizol (Invitrogen) extracted, Oregon R embryonic cRNA that was oligo-dT primed and reverse transcribed by Superscript III (Invitrogen). The isoform RA clone obtained differs from FlyBase annotations in 2 locations where either a downstream splice site was used, as in isoforms B, D, and E, resulting in 12 extra amino acids and an additional unannotated exon was included resulting in an additional 11 amino acids; RRM domains remain intact in this isoform A variant. The *shep*, *su(Hw)*, and *mod(mdg4)2.2* cDNAs were inserted into pENTR/D-TOPO and recombined into pDEST 15 (Invitrogen) to generate N-terminal GST fusion constructs. All plasmids were sequenced for verification.

### Recombinant protein and GST-pulldown

Expression of GST, GST-Shep, GST-Su(Hw), GST-Mod(mdg4)2.2, His-Su(Hw), and His-Mod(mdg4)2.2 was induced in *E. coli BL21* cells by 1 mM Isopropyl β-D-1-thiogalactopyranoside at 37° C. Proteins were purified under native conditions by affinity using Glutathione-Agarose (Pierce) or Ni-NTA-Agarose (Qiagen). 2 µg immobilized GST or GST fusion proteins were incubated with 35 µg soluble His-Mod(mdg4)2.2 in PBSMT (137 mM NaCl, 2.7 mM KCl, 10 mM NaH_2_PO_4_, 1.8 mM KH_2_PO_4_, 250 mM MgCl_2_, 0.3% Triton X-100 supplemented with Complete protease inhibitors (Roche), 1 mM PMSF, 1 mM DTT, and 100 mg/mL BSA) in a volume of 350 µL. Binding reactions were carried out at 4°C rotating for 2 h. Unbound protein was removed, and beads were washed 5 times in PBSMT. Bound His-Mod(mdg4)2.2 was eluted in sample buffer, separated by SDS-PAGE, and detected by Western blotting. His-Su(Hw) binding reactions were carried out in the same manner except in HBSM (50 mM HEPES, pH 6.7; 150 mM NaCl; 5 mM KCl; 2.5 mM MgCl_2_) supplemented with 0.3% Triton-X 100, 0.2 M KCl, protease inhibitors, 1 mM PMSF, 1 mM DTT, and 100 mg/mL BSA.

### Antibodies and antibody production

6X-His-Shep isoform E (AA169–368), CP190 antigen [4], Su(Hw) antigen [41] and Mod(mdg4)2.2 antigen [47] were expressed in BL21 cells, affinity purified by Ni-NTA-agarose (Qiagen) according to the manufacturer's protocol under denaturing conditions and used to immunize rabbits and guinea pigs using standard procedures (Covance Research Products). For Western blotting, guinea pig α-Shep serum was used at 1∶2000, guinea pig α-CP190 was used at 1∶10,000, guinea pig α-Mod(mdg4)2.2 [41] was used at 1∶1000, guinea pig α-Su(Hw) [41] was used at 1∶7500, α-Pc [32] was used at 1∶1000, α-E(z) [48] was used at 1∶1000, and α-Pep [49] was used at 1∶1000. For insulator body staining, rabbit α-CP190 [4] was used at 1∶30,000. The monoclonal α-Elav9AF89 was obtained from the Developmental Studies Hybridoma Bank and used at 1∶1000, and guinea pig α-Shep serum was used at 1∶200 for IF.

### Coimmunoprecipitation

Nuclei from 20 g of embryos were prepared as described [20]. Nuclei were lysed in 4 mL HBSM supplemented with 0.3% TritonX-100 (HBSMT), complete protease inhibitors and 1 mM PMSF by dounce homogenization with the B pestle. Extracts were cleared of insoluble material by centrifugation, and half of the supernatant was incubated with pre-immune serum and half with α-Shep serum pre-conjugated to protein A sepharose. IPs were carried out for 1 h at 4°C, rotating. Unbound protein was removed and beads were washed 4 X in HBSMT and 1 X in HBSM. Bound protein was eluted in sample buffer, separated by SDS-PAGE, and detected by Western blotting.

### Immunofluorescence

Polytene chromosome spreads were prepared as described previously [8]. Brains and imaginal discs were dissected from at least 5 larvae of each genotype per experiment and stained as described previously [32]. Chromosomes and discs were imaged using a Leica DM5000B epifluorescent microscope and captured using OpenLab software.

Indirect immunofluorescence of mixed stage Oregon R embryos was carried out as described [50], [51]. Blocked embryos were incubated rotating with primary antibodies overnight at 4°C and secondary antibodies for 2 h at 37°C. After washing, embryos were incubated in DAPI and mounted in 2.5% DABCO (Sigma) in 70% glycerol. Embryos were imaged on a Zeiss 510 confocal microscope.

### Luciferase insulator assay

Females homozygous for *attP3*::UAS-luciferase transgenes were crossed to Gal4 expressing males; luciferase in individual F1 male larvae was quantified. Any homozygous lethal Gal4 lines were selected against GFP expressing balancer chromosomes. Larvae were collected on dry ice and stored at −80°C until use, at which time they were homogenized in 30 µL Glo Lysis buffer (Promega) and incubated at room temperature for 10 min. Debris was cleared from extracts by centrifugation, and 20 µL soluble material was dispensed into opaque 96-well plates; the same volume of luciferase reagent (Promega) was added to each well, and plates were incubated in the dark for 10 min. Light emission was quantified using a Spectramax II Gemini EM plate reader (Molecular Devices). Luciferase values were normalized to total protein determined by Bradford assay carried out in parallel. Luciferase values between genotype populations were log transformed to obtain a normal distribution and compared by one-way ANOVA. Tukey HSD *post hoc* tests were used to determine pairwise *p* values between genotypes. For further information including additional Gal4 lines tested, see Text S1.

### Cell culture

BG3-c2 cells were grown in S2 medium (Sigma) supplemented with 10% fetal calf serum and 10 µg/mL insulin. Cells were maintained in monolayer at 25°C.

### Chromatin immunoprecipitation and ChIP–seq library construction

Cells were fixed in 1% formaldehyde added directly to cells in culture medium for 10 min at RT with gentle agitation; formaldehyde was quenched by addition of glycine to 0.125 M with gentle agitation for 5 min at RT. 5×10^6^ to 10^7^ cells were used per IP. Cells were pelleted at 400 rcf and washed twice in ice cold PBS. Cells were resuspended in 1 mL ice cold cell lysis buffer (5 mM PIPES, pH 8, 85 mM KCl, 0.5% NP-40) supplemented with protease inhibitors, and nuclei were released by Dounce homogenization with the B pestle and pelleted by centrifugation at 9190 rcf for 5 min at 4°C. Nuclei and chromatin were further processed as described [41]. Chromatin was fragmented to an average size of 300 bp by sonication and validated by agarose gel electrophoresis. Sequencing libraries were prepared according to the standard Illumina ChIP-seq protocol. Highly similar profiles were obtained with two independent α-Shep antibodies; therefore, the antibody (guinea pig) displaying the highest signal to noise ratio was utilized for subsequent analyses. Rabbit α-Su(Hw) [16] and rabbit α-Mod(mdg4)2.2 [42] were used for ChIP-seq. Highly similar profiles were obtained with two independent α-Mod(mdg4)2.2 antibodies [41]; therefore, the antibody displaying the highest signal to noise ratio was utilized for subsequent analyses. Libraries were constructed with TruSeq adapters and sequenced on an Illumina HiSeq multiplexed in a single lane. For directed ChIP, quantitative PCR was performed as previously reported [32].

### Computational analyses

#### ChIP–seq

36 bp reads from the Illumina HiSeq 2000 sequencer were mapped to dm3 chromosomes except chrUextra, using Bowtie v0.12.7 with parameters “–best –strata -m1 -n2 –tryhard”. Reads from repetitive regions were removed, and duplicates were removed with MarkDuplicates from Picard 1.49. Peak-calling was performed with SPP [43] using default parameters (e.g., FDR = 0.01, z-threshold = 3) with the exception of “srange = c(50, 200)” when calculating binding site characteristics to improve symmetry of the auto-correlation curve. Broad peak regions were added to binding site point positions, and final peaks were merged. Sequence data are deposited in the Gene Expression Omnibus under accession number GSE40797.

#### Downstream analyses

Intersections, classification, and randomizations in the below analyses were performed with pybedtools v0.6 [52], gffutils v0.8, and BEDTools v2.16.2 [53].

#### Pie charts

Feature classes [TSSs (1 bp transcript start position), CDSs, introns, 5′UTRs, and 3′UTRs] were extracted from all annotated isoforms of all annotated genes in FlyBase release 5.33. Intergenic regions were defined as the remainder of dm3. Since a ChIP-seq peak can fall in more than one class, we classified a peak by its highest priority annotation class, where the priorities from highest to lowest are TSS, CDS, intron, 5′UTR, 3′UTR, and intergenic.

#### Heat maps

Data files containing called peaks were downloaded from GEO and modENCODE [54]–[56] and converted to BED files. Shep peaks that overlapped either a Su(Hw) peak or a Mod(mdg4)2.2 peak by at least one base were filtered out to create a set of non-*gypsy* Shep peaks. Enrichment scores were calculated as follows: For each pairwise comparison between files A and B, the Jaccard statistic (intersection of bp divided by union of bp; as described previously [57]) was computed to obtain the “actual” statistic. Then, features in file A were shuffled to a random position on the same chromosome, and the Jaccard statistic was again calculated. After 1000 such shufflings, the actual statistic was divided by the median of the empirical distribution to get an enrichment score, (actual+1)/(median randomized+1), for the comparison. The full enrichment matrix was hierarchically clustered using correlation as a distance metric and complete linkage clustering as implemented in SciPy, with rows clustered identically as columns. Selected rows from the full clustered matrix in Figure S4 are shown in Figure 6D.

#### Colocalization of Su(Hw), Mod(mdg4)2.2, and Shep

To assess the possibility of Shep and Mod(mdg4)2.2 binding mutually exclusively to Su(Hw) sites, we created an N×M binary matrix of binding sites where N = 8194 is the number of binding sites containing any of Su(Hw), Shep, or Mod(mdg4)2.2 (using the pybedtools.contrib.plotting.binary_heatmap() function) and M = 3 for the three factors. We then took the set of 1356 Su(Hw) sites with Shep, Mod(mdg4)2.2, or both, and extracted the Shep and Mod(mdg4)2.2 vectors for these sites representing a total of 663 Shep+Su(Hw) and 964 Mod(mdg4)2.2+Su(Hw) sites. There were 271 Shep+Mod(mdg4)2.2 colocalization events in these vectors. We then randomly shuffled the vectors 10,000 times, computing colocalization each time, and obtained a mean of 472 colocalization events with no iteration giving less than 438 colocalization events. Therefore, of the Su(Hw) sites containing either Shep or Mod(mdg4)2.2, the actual Shep+Mod(mdg4)2.2 overlap of 271 suggests Shep, Mod(mdg4)2.2, and Su(Hw) colocalize significantly less often than expected (empirical *p*<1×10^−4^). These results are also consistent with a hypergeometric test using n = 1356, n1 = 663, n2 = 964, and m = 271 (*p* = 2.2×10^−16^).

The same analysis was performed for 1403 Mod(mdg4)2.2 sites with Su(Hw), Shep, or both as well as 964 Mod(mdg4)2.2+Su(Hw) and 710 Mod(mdg4)2.2+Shep extracted sites. The mean of randomized iterations was 488 and none had less than 454 colocalization events (empirical *p*<1×10^−4^) and (*p* = 2.2×10^−16^, hypergeometric test).

For 1102 Shep sites with Su(Hw), Mod(mdg4)2.2, or both as well as 710 Shep+Mod(mdg4)2.2 and 663 Shep+Su(Hw) extracted sites. The mean of randomized iterations was 427 and none had less than 393 colocalization events (empirical *p*<1×10^−4^) and (*p* = 2.2×10^−16^, hypergeometric test).

## Supporting Information

Figure S1 Specific coimmunoprecipitation of *gypsy* insulator proteins with Shep. Embryo nuclear extracts (lane 1) were immunoprecipitated (IP) with either Pre-Immune (Pre Im; lanes 2 and 4) or α-Shep (lanes 3 and 5) serum. Shep, Mod(mdg4)2.2, Su(Hw), and CP190 were detected in nuclear extracts (Nuc Ext), supernatants (Sup, lanes 4–5) and IPs (lanes 2–3) by Western blotting. The nuclear proteins E(z) and Pc were used as negative controls for Shep IP.
(TIF)

Figure S2 The *attP3* landing site is located in a PcG repressed region. H3K27me3 and PhoL ChIP-chip signal from embryos at the location of *attP3* on the X chromosome [58].
(TIF)

Figure S3 Immunolocalization of Shep on polytene chromosomes. (A) Localization of Shep and each *gypsy* insulator protein as indicated on larval salivary gland polytene chromosomes. Guinea pig α-Shep (red) was detected with α-guinea pig conjugated Alexa-594. Rabbit α-Su(Hw), α-Mod(mdg4)2.2, or α-CP190 (green) were detected with α-rabbit conjugated Alexa-488. DAPI stained DNA (blue) is shown in the merged image. White arrow indicates the presence of Shep on a highly transcribed puff region. (B) Localization of Shep and Su(Hw) at a band/interband boundary. Yellow arrow indicates a band/interband boundary where both Shep and Su(Hw) colocalize. DAPI is shown in blue in the merge.
(TIF)

Figure S4 Full heat map of pairwise comparisons of binding sites for a particular factor and hierarchical clustering. Pairwise comparisons of binding sites for a particular factor as in Figure 6D with hierarchical clustering. Rows are clustered by complete linkage using correlation as the distance metric, and columns are sorted identically to rows.
(TIF)

Table S1 Number of flies scored for *ct^6^* phenotype for each genotype reported in Figure 2 and Table 1.
(DOC)

Table S2 Primers used.
(DOC)

Text S1 Development of the luciferase barrier assay.
(DOC)
