# Supplementary figures and images for: Tissue-Specific Regulation of Chromatin Insulator Function
Source: PLoS Genet. 2012 Nov 29;8(11):e1003069. doi: 10.1371/journal.pgen.1003069 (PMC3510032; doi:10.1371/journal.pgen.1003069)

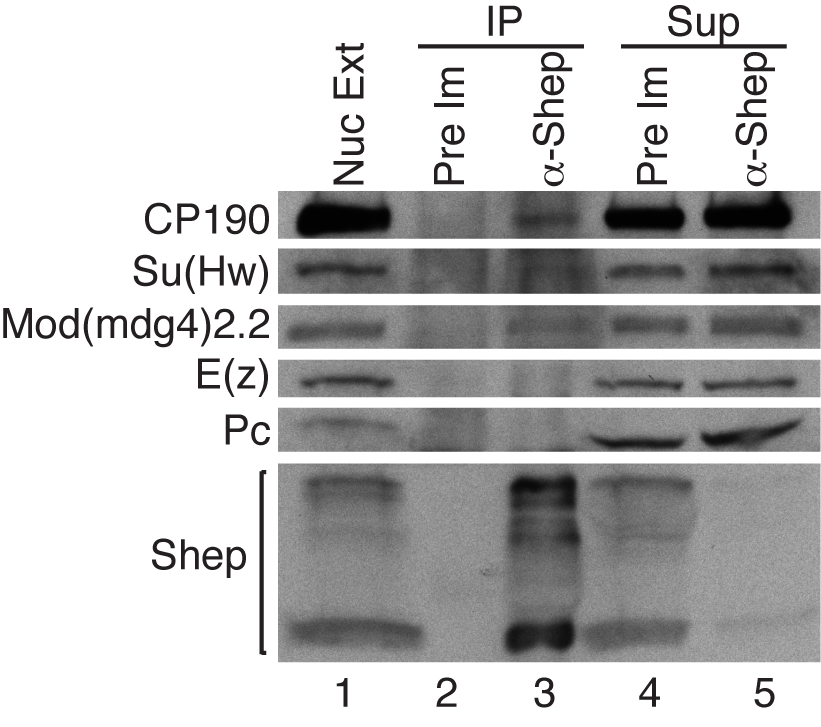

Supplement: Figure S1 — Specific coimmunoprecipitation of gypsy insulator proteins with Shep. Embryo nuclear extracts (lane 1) were immunoprecipitated (IP) with either Pre-Immune (Pre Im; lanes 2 and 4) or α-Shep (lanes 3 and 5) serum. Shep, Mod(mdg4)2.2, Su(Hw), and CP190 were detected in nuclear extracts (Nuc Ext), supernatants (Sup, lanes 4–5) and IPs (lanes 2–3) by Western blotting. The nuclear proteins E(z) and Pc were used as negative controls for Shep IP. (TIF) [file pgen.1003069.s001.tif]

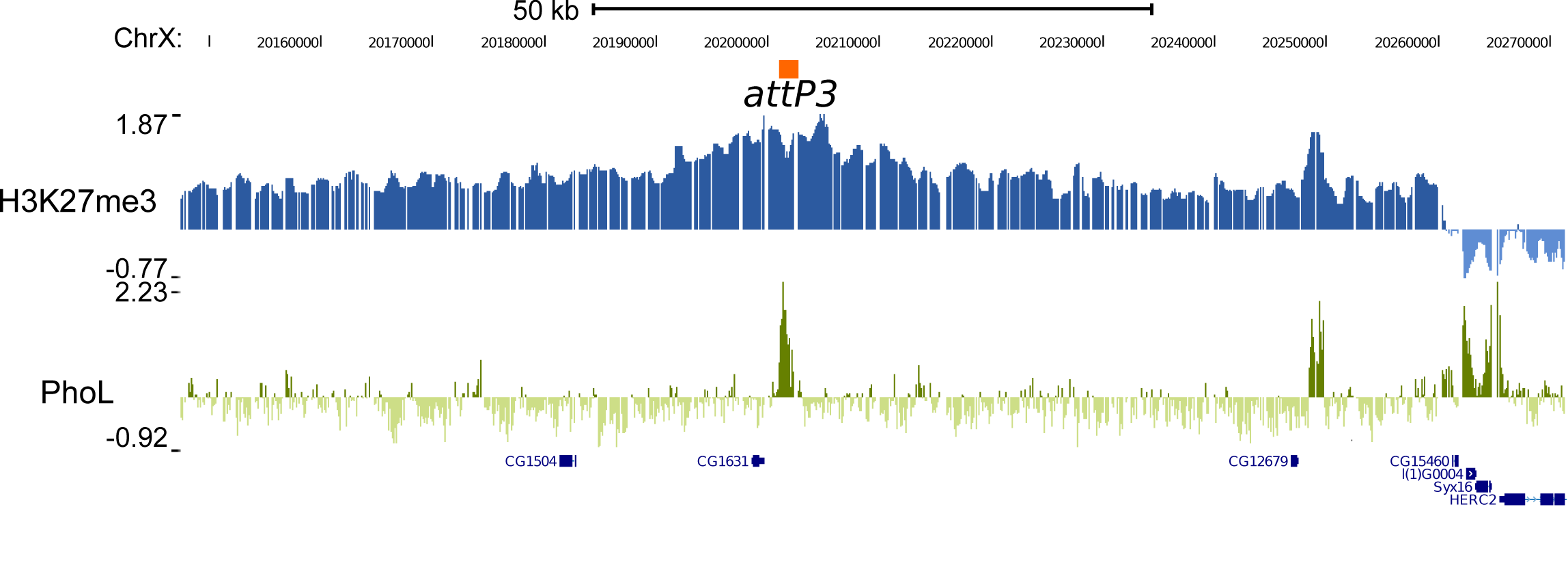

Supplement: Figure S2 — The attP3 landing site is located in a PcG repressed region. H3K27me3 and PhoL ChIP-chip signal from embryos at the location of attP3 on the X chromosome [58]. (TIF) [file pgen.1003069.s002.tif]

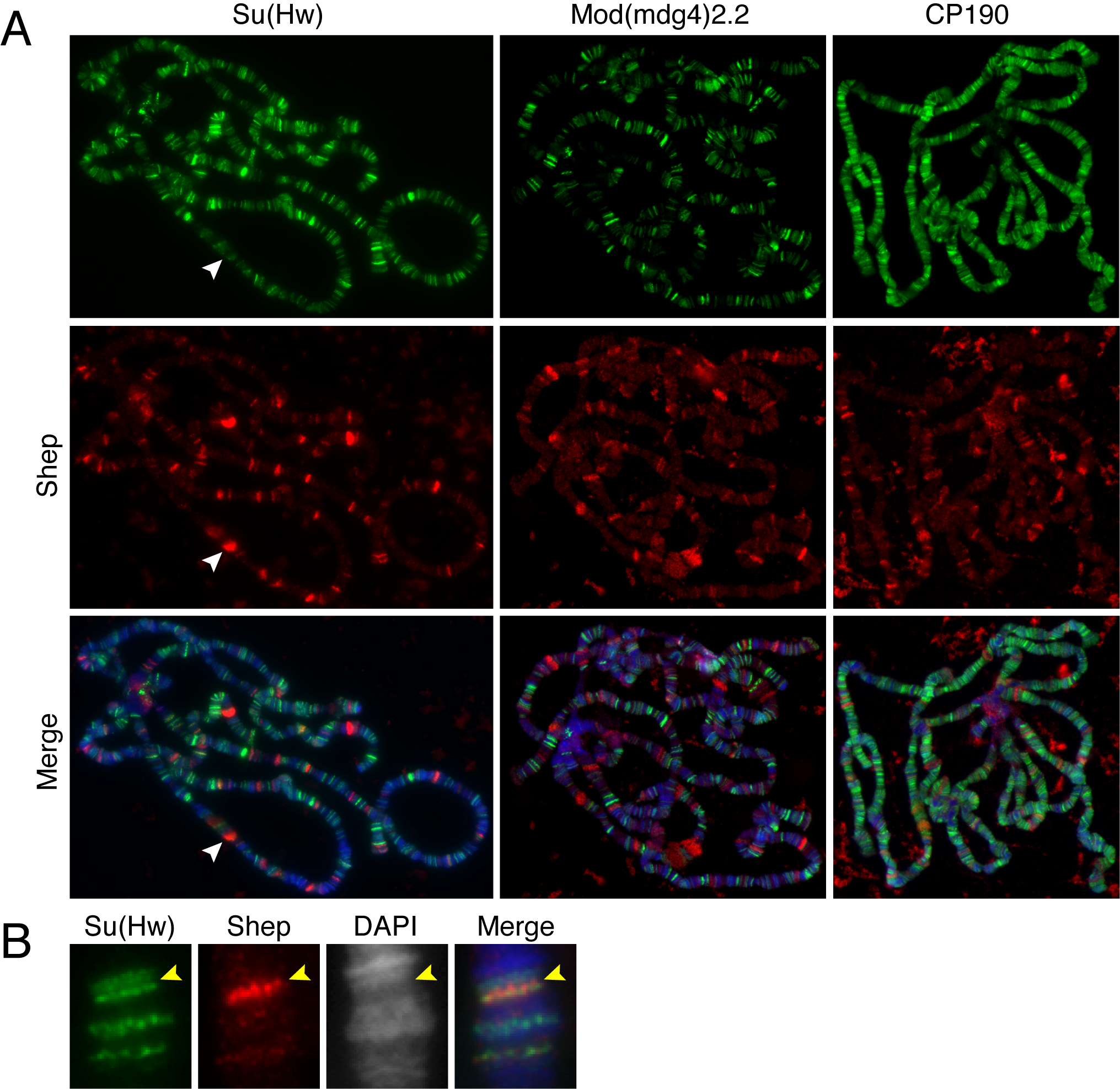

Supplement: Figure S3 — Immunolocalization of Shep on polytene chromosomes. (A) Localization of Shep and each gypsy insulator protein as indicated on larval salivary gland polytene chromosomes. Guinea pig α-Shep (red) was detected with α-guinea pig conjugated Alexa-594. Rabbit α-Su(Hw), α-Mod(mdg4)2.2, or α-CP190 (green) were detected with α-rabbit conjugated Alexa-488. DAPI stained DNA (blue) is shown in the merged image. White arrow indicates the presence of Shep on a highly transcribed puff region. (B) Localization of Shep and Su(Hw) at a band/interband boundary. Yellow arrow indicates a band/interband boundary where both Shep and Su(Hw) colocalize. DAPI is shown in blue in the merge. (TIF) [file pgen.1003069.s003.tif]

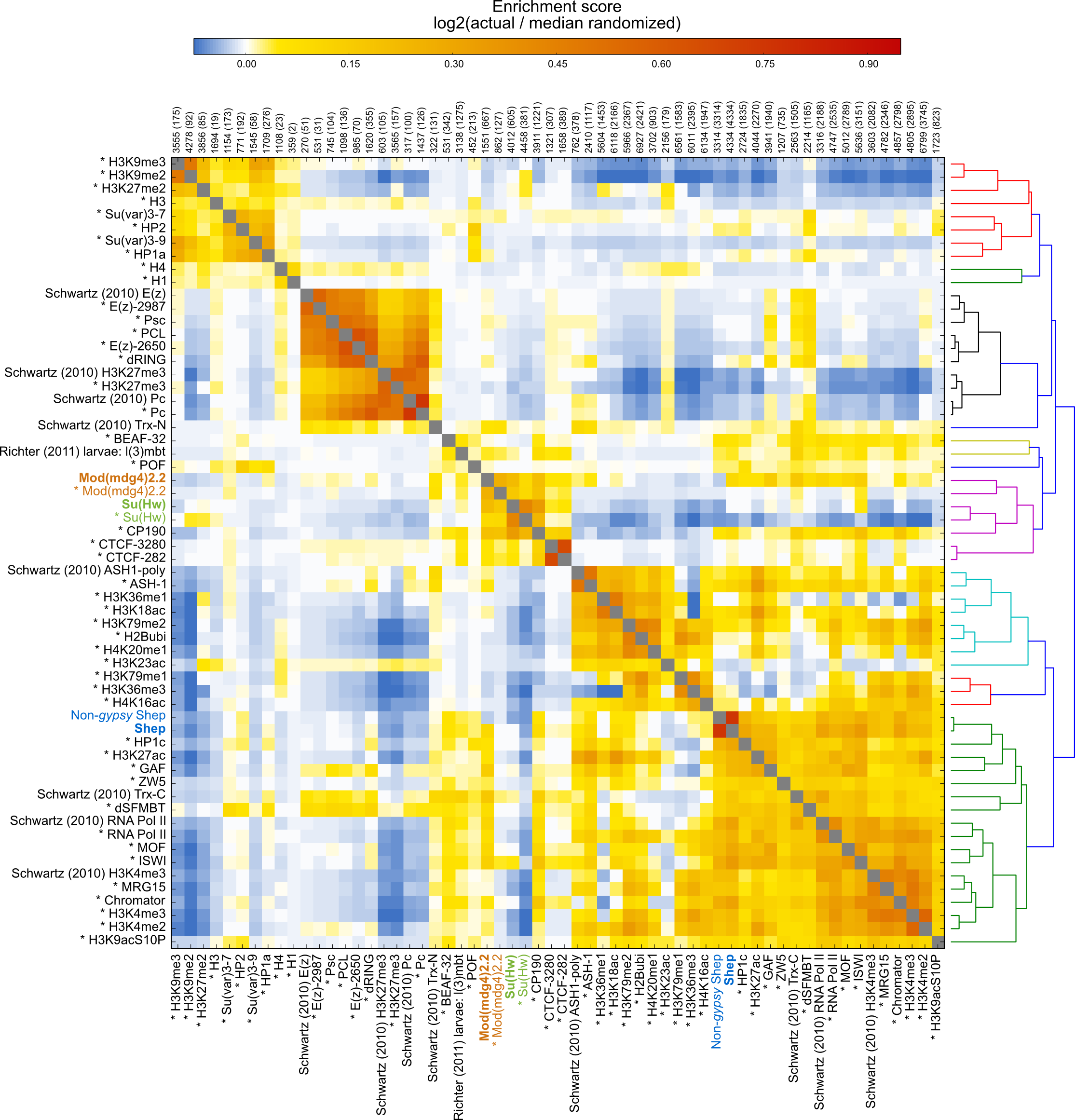

Supplement: Figure S4 — Full heat map of pairwise comparisons of binding sites for a particular factor and hierarchical clustering. Pairwise comparisons of binding sites for a particular factor as in Figure 6D with hierarchical clustering. Rows are clustered by complete linkage using correlation as the distance metric, and columns are sorted identically to rows. (TIF) [file pgen.1003069.s004.tif]
