# Supplementary material for: Tissue-Specific Regulation of Chromatin Insulator Function
Source: PLoS Genet. 2012 Nov 29;8(11):e1003069. doi: 10.1371/journal.pgen.1003069 (PMC3510032; doi:10.1371/journal.pgen.1003069)
Supplement: Table S1 — Number of flies scored for ct6 phenotype for each genotype reported in Figure 2 and Table 1. (DOC) [file pgen.1003069.s005.doc]

|  | ***ct^6^*** | | | | **Synthetic lethality** | | |
| --- | --- | --- | --- | --- | --- | --- | --- |
| **P-element name** | Homo-zygous | With *Df(3L)ED210* | With *Df(3L)Exel6103* | With *Df(3L)Exel6104* | Live homo-zygotes | Dead homo-zygous pupae | Empty homozygous pupal cases |
| *+* (none) | 180 | 61 | 144 | 116 | 67 | 0 | 75 |
| *BG00836* | 51 | 12 | 12 | 101 | 7 | 22 | 54 |
| *d05714* | 49 | 12 | 31 | 85 | 30 | 49 | 82 |
| *d04425* | 148 | 68 | 124 | 124 | 117 | 0 | 113 |
| *EY04794* | 125 | 64 | 110 | 113 | NQ | NQ | NQ |
| *DG30207* | 137 | 86 | 86 | 160 | NQ | NQ | NQ |
| *KG10149* | 58 | 60 | 92 | 111 | 56 | 6 | 83 |
| *e00306* | 100 | 98 | 87 | 93 | 67 | 2 | 83 |
| *BG02468* | 137 | 86 | 74 | 154 | NQ | NQ | NQ |
| *BG02613* | 78 | 65 | 130 | 173 | 75 | 2 | 130 |
| *BG00655a* | 114 | 79 | 42 | 130 | 71 | 2 | 109 |

**Table S1. Number of flies scored for *ct^6^* phenotype reported in Figure 3 and Table 1 and synthetic lethality reported in Table 1.**

Number of flies scored (*n*) for each *shep* P-element insertion as homozygous or transheterozygous with genomic deficiencies as indicated; NQ, not quantified; % survival in Table S1 is the percent of live homozygotes with respect to the sum of dead and eclosed homozygous pupae.
