# Supplementary material for: Tissue-Specific Regulation of Chromatin Insulator Function
Source: PLoS Genet. 2012 Nov 29;8(11):e1003069. doi: 10.1371/journal.pgen.1003069 (PMC3510032; doi:10.1371/journal.pgen.1003069)
Supplement: Table S2 — Primers used. (DOC) [file pgen.1003069.s006.doc]

**Table S2. Primers Used**

| **Cloning** | **Name** | **Sequence** |
| --- | --- | --- |
|  | 5' shep antigen | CACCtttgccgatggcggaccg |
|  | 3' shep+stop | ctactatttgggagcggcttg |
|  | 5' shep A | cacccacccacgatacagcccag |
|  | 5' shep C and E | CACCggtcccaacggcacgg |
|  | 5' shep B/D | caccggccagacaagtcccgcagc |
|  | 5’ Shep E pUASTattb | GCGGCCGCATGggtcccaacggcacgg |
|  | 3’ Shep E pUASTattB | TCTAGActatttgggagcggcttg |
|  | 5’ RNP1 mutation | gaCGGCgaCGTCGACgaCGAGCAGCCAGCCTTCGCCGAGTG |
|  | 3’ RNP1 mutation | GtcGTCGACGtcGCCGtcACCTTTACATTTGTTTGTTGTTTTATC |
|  | 5’ RNP2 mutation | GcTGGCgcTGCCgcCATGGAGAGTCGCGAGAAGTGCGAGCAAATC |
|  | 3’ RNP2 mutation | GgcGGCAgcGCCAgCGCCCTTGGAGTTCATCTGCTGATCAC |
