# Supplementary material for: Tissue-Specific Regulation of Chromatin Insulator Function
Source: PLoS Genet. 2012 Nov 29;8(11):e1003069. doi: 10.1371/journal.pgen.1003069 (PMC3510032; doi:10.1371/journal.pgen.1003069)
Supplement: Text S1 — Development of the luciferase barrier assay. (DOC) [file pgen.1003069.s007.doc]

**Text S1 for**

**Tissue-specific regulation of chromatin insulator function**

Leah H. Matzat, Ryan K. Dale, Nellie Moshkovich, Elissa P. Lei

**Development of the luciferase barrier assay**

Our data demonstrate a novel and versatile *gypsy* barrier assay as a powerful tool to assess tissue-specific insulator activity. We identified two caveats, however, that limit utility of the assay. First, luciferase activity must be sufficiently high to detect changes in insulator activity. We found that several specialized CNS Gal4 drivers tested, *elav*::Gal4, *ap*::Gal4, *nrv2*::Gal4, and *sim::*Gal4, exhibited luciferase levels below which quantifiable changes in activity are detectable (data not shown). Furthermore, although the *attP3* site is silenced in the CNS and in muscle tissue (Figures 5F and G), luciferase is highly activated by *GMR*::Gal4 in non-insulated lines (data not shown), indicating that not all tissues are PcG repressed at this genomic location. These data show that the *attP3* luciferase reporter is useful in the majority of tissues, including the CNS and muscle; the growing library of *attP* insertion sites might be utilized to identify additional barrier sites useful in other tissues.
